# Association Between Physical Activity, Quality of Life, Barriers to Physical Activity, and Mental Health in Chilean Adolescents: The MOV-ES Study

**DOI:** 10.3390/healthcare13162028

**Published:** 2025-08-17

**Authors:** Eugenio Merellano-Navarro, Pablo Pasten-Hernández, Juan Aristegui-Mondaca, Antonia Morán-Toloza, Marcelo Nuñez-Galaz, Frano Giakoni-Ramírez, Daniel Duclos-Bastías, Andrés Godoy-Cumillaf

**Affiliations:** 1 Department of Physical Activity Sciences, Faculty of Education Sciences, Universidad Católica del Maule, Talca 3530000, Chile; 2 Centro Integral Educación, Talca 3461380, Chile; pablo.pasten@alu.ucm.cl; 3 Master Program of Physical Activity Sciences, Faculty of Education Sciences, Universidad Católica del Maule, Talca 3530000, Chile; juan.aristegui@alu.ucm.cl (J.A.-M.); antonia.moran@alu.ucm.cl (A.M.-T.); 4 Municipalidad de Lolol, Región del Libertador General Bernardo, O’Higgins 3140000, Chile; marcelonunez@lololeduca.cl; 5 Facultad de Educación y Ciencias Sociales, Instituto del Deporte y Bienestar, Universidad Andrés Bello, Santiago 7550000, Chile; frano.giakoni@unab.cl; 6 Escuela de Educación Física, Pontificia Universidad Católica de Valparaíso, Valparaíso 2340000, Chile; daniel.duclos@pucv.cl; 7 Grupo de Investigación en Educación Física, Salud y Calidad de Vida (EFISAL), Facultad de Educación, Universidad Autónoma de Chile, Temuco 4780000, Chile

**Keywords:** mental health, physical activity, health-related quality of life, adolescents

## Abstract

Objective: To analyze the association between physical activity, health-related quality of life (HRQoL), and perceived barriers to physical activity with the risk of symptoms of depression, anxiety, and stress in Chilean adolescents. Method: A quantitative, cross-sectional, descriptive-correlational study was conducted with a sample of 351 secondary school students (mean age = 15.75 ± 1.47 years) from several educational institutions in the south-central region of Chile. Validated instruments were used to assess physical activity (PAQ-A), symptoms of mental health (DASS-21), HRQoL (Kidscreen-52), and the short scale of barriers to physical activity. For exploratory purposes, mental health outcomes were dichotomized based on standard cut-off scores, and binary logistic regression models were estimated to identify associated factors. Results: Based on the binary categorization, a substantial proportion of students exceeded the risk thresholds for depressive (54.4%), anxiety (63%), and stress symptoms (42.2%). Across models, lower physical activity levels, reduced autonomy and weaker relationships with parents, and barriers related to self-concept and motivation were consistently associated with higher mental health risk. Additionally, passive commuting and the perceived school environment emerged as specific predictors of stress and depression risk, respectively. Conclusions: These findings suggest that individual and contextual factors linked to lifestyle behaviors and perceived social support may play a critical role in adolescent mental health, and could represent key targets for school-based interventions.

## 1. Introduction

Adolescence is a critical stage in human development, characterized by profound physical, emotional, and social changes [1]. During this period, the lifestyle habits acquired can have a substantial impact on future health, particularly in domains such as health-related quality of life (HRQoL), physical activity, and mental health [2,3].

Currently, the mental health of children and adolescents has become a critical public health challenge worldwide [4]. The relevance of mental health in young people transcends the individual level, impacting the social and economic fabric of societies [5]. Therefore, early and effective interventions can prevent the progression of mental disorders and reduce the risk of chronic problems in adulthood [6]. In Chile, recent research has revealed a high prevalence of mental health problems among adolescents (60.2% with symptoms of depression, 63.6% with anxiety, and 50.2% with stress) [4]. Another national study reported that 83.6% of adolescents (mean age 16 years) presented symptoms of depression and 88.5% presented symptoms of anxiety [7]. While these findings highlight the urgency of addressing adolescent mental health as a priority public health issue, it is important to consider that such high prevalence rates may be influenced by methodological aspects, such as the measurement instruments used, sampling strategies, or cut-off points applied, as well as by contextual factors such as socioeconomic inequality, healthcare accessibility, exposure to violence, the built environment where they live, academic pressure, and the lingering psychosocial effects of the COVID-19 pandemic.

Furthermore, evidence indicates a worrying increase in sedentary behavior and a decrease in physical activity levels among adolescents [8], which, combined with socioeconomic and academic barriers, negatively affect their mental health and overall well-being [9,10]. Studies that have explored barriers to physical activity in this age group have identified motivation, social influences, parental support, screen time, and the built environment as particularly relevant factors [11,12,13,14]. These findings underscore the need to design and implement strategies that promote physical activity to improve both physical and mental health in adolescents [15], which in turn can contribute to better sexual and reproductive health outcomes [16,17].

The HRQoL is a multidimensional construct that encompasses physical, emotional, mental, social, cultural, and behavioral aspects of well-being and functioning as perceived by individuals [18,19]. Although physical activity is known to influence HRQoL and mental health [17], there are gaps in the literature regarding its actual impact. Most studies in this field have employed weak methodological designs, limiting the possibility of establishing causal relationships between these variables [20].

Previous studies in this area have often been limited by small or non-representative samples, the use of non-validated measurement instruments, and the omission of relevant contextual factors such as socioeconomic status, environmental characteristics, and perceived barriers to physical activity [21]. Moreover, few investigations have simultaneously examined the combined influence of physical activity, HRQoL, and perceived barriers on mental health outcomes in adolescents [22,23]. Although the present study also adopts a cross-sectional design, it addresses some of these limitations by including a large and diverse sample of Chilean adolescents, employing standardized and validated instruments, and analyzing multiple interrelated variables within a single analytical framework. For these reasons, the present study aims to analyze the association between physical activity, HRQoL, and perceived barriers to physical activity as predictors of the risk of symptoms of depression, anxiety, and stress in Chilean adolescents.

## 2. Method

This study forms part of the second phase of a larger project aimed at evaluating the effect of an active transportation education program, based on the ecological model on improving the physical and mental health of secondary school students [24]. The current phase employed a quantitative, cross-sectional design with a descriptive-correlational scope. The target population consisted of secondary school students from various educational institutions in the central region of Chile.

### 2.1. Participants

The sample was a non-probabilistic convenience sample and included 351 adolescents (48% male and 52% females), with a mean age of 15.75 ± 1.47 years, from urban (48%) and rural (52%) areas. The inclusion criteria were as follows: (i) actively enrolled in secondary education at one of the invited schools, (ii) attendance on the day of instrument administration, and (iii) provision of informed consent. Incomplete questionnaires were considered an exclusion criterion. A final sample comprising 351 adolescents (48% male and 52% female) was obtained, with a mean age of 15.75 ± 1.47, belonging to schools located in rural (52%) and urban (48%) areas. The study was approved by the ethics committee of Universidad Católica del Maule (Code: 27/2024).

### 2.2. Procedures

The research team implemented several strategies to encourage adolescent participation. First, the purpose of the study was communicated via email and/or in person to the administrators of each institution, requesting authorization to invite adolescents during physical education class time. Subsequently, an invitation was sent to the adolescents’ parents, including consent forms to be signed and returned via their children on the day of evaluation. Once authorization was granted, the team attended the class, presented the objectives using audiovisual materials, and invited the adolescents to complete the instruments. The time allocated to complete all questionnaires was 20 min. All evaluations were conducted during April and May 2025.

### 2.3. Instruments

A series of questionnaires was administered, including instruments validated for adolescent populations, covering areas such as sociodemographic information, mental health, physical activity, health-related quality of life, and barriers to physical activity. The instruments were administered by final-year students in the Physical Education Teaching degree program who had been trained in their use, during class hours assigned by each school, always in the presence of teachers from each school.

Sociodemographic information: data such as name, age, gender, nationality, type of school, area of residence, and sports practice were requested.

Physical activity: The Physical Activity Questionnaire for Adolescents (PAQ-A) was completed. The self-report scale, described by its creators as designed for use with scalar students, contains eight items intended to capture adolescents’ recall of their physical activity during the previous seven days. The final score is calculated as the arithmetic mean of the scores from the eight questions. Question 9 assesses whether the adolescent was ill or experienced any circumstance that prevented them from engaging in physical activity during that week. The PAQ-A has demonstrated good reliability and validity in diverse adolescent populations [25].

Barriers to physical activity: The short scale for perceiving barriers to physical activity in adolescents was used. This is a twelve-item self-report instrument that asks adolescents to state the extent to which they perceive the different items included in the scale as barriers to participating in organized sports activities. Each item was evaluated using a five-point Likert scale, where 1 means strongly disagree and 5 means strongly agree. The self-concept subscale was formed with the mean scores of questions 4, 6, 8, and 11, the motivation and interest subscale with questions 5, 7, 10, and 12, the social support subscale with questions 3 and 9, and finally, the task incompatibility subscale with questions 1 and 2. The higher the score on each subscale, the greater the barrier to physical activity. This scale has demonstrated acceptable psychometric properties in adolescent populations [26].

Health-related quality of life: This was obtained using the Kidscreen-52, which measures the attributes of ten dimensions of health: physical well-being, psychological well-being, moods and emotions, self-perception, autonomy, relationships with parents and home life, financial resources, peers and social support, school environment, and bullying. Each dimension is made up of a series of questions that together give a scale ranging from 0 (the worst health state for that dimension) to 100 (the best health state). The Kidscreen-52 has been validated in various adolescent populations worldwide, including Chile [27].

Mental health: The Depression, Anxiety, and Stress Scale (DASS-21) was used. It is an instrument designed to assess the negative emotional states of depression, anxiety, and stress. The depression subscale is formed by questions 3, 5, 10, 13, 16, 17, and 21, questions 2, 4, 7, 9, 15, 19, and 20 form the anxiety subscale, and the stress subscale is made up of questions 1, 6, 8, 11, 12, 14, and 18. Participants were then categorized into “no risk” or “risk” groups based on established cut-off scores validated in adolescent populations: depression ≥ 5, anxiety ≥ 4, and stress ≥ 8. This binary classification approach has been widely used in epidemiological studies with adolescents to facilitate prevalence estimation and group comparisons [28]. The DASS-21 has been widely validated internationally, including in adolescent populations [29,30].

### 2.4. Statistical Analysis

Statistical analysis was performed using IBM SPSS Statistics software version 27.0.1. The normality of the sample was assessed using the Kolmogorov–Smirnov test. The descriptive characteristics of the sample were presented using measures of central tendency and dispersion (mean ± standard deviation) for continuous variables, compared using Student’s *t*-test. The effect size was calculated using Cohen’s d. For categorical variables, frequency distributions were used and the chi-square test was applied. A statistical significance level of *p* < 0.05 was established. Additionally, three binary logistic regression analyses were performed, considering the risk of depression, risk of anxiety, and risk of stress as dependent variables. The independent variables included all dimensions of health-related quality of life, barriers to physical activity, physical activity score, as well as gender, geographical location, type of transportation to school, and age. The final model was determined using the backward selection method based on the Wald statistic. The goodness of fit of the binary logistic regression models was evaluated using the Hosmer–Lemeshow test. Multicollinearity among the independent variables was also examined using the variance inflation factor (VIF), considering a VIF threshold < 5 as an indicator of the absence of significant collinearity among predictors.

## 3. Results

Table 1 presents the characteristics of the sample. Of all participants, 52% are women and 66.1% report practicing a sport. A total of 51.6% of the sample belongs to the rural area. The mean PAQ-A score was 2.26 ± 0.77, being higher in men (*p* = 0.000). Regarding the frequency of risk of depression, adolescent girls have a 10.9% higher risk than men, 10.1% for anxiety, and 10% for stress. In relation to HRQoL, men have higher scores in each dimension, all of which are significant except for social support and peers, and only in physical well-being is Cohen’s d high. The results show higher scores for barriers to physical activity in women, with statistically significant differences in self-concept and motivation and interest, and a low Cohen’s d.

Table 2 shows the HRQoL and barriers to physical activity scores based on the risk of depression, anxiety, and stress. Higher scores are observed in all HRQoL dimensions and lower scores in barriers in subjects who do not present a mental health risk. Social and peer support and physical activity scores did not show significant differences in any of the mental health variables. Only task incompatibility was not statistically significant in depression.

Table 3, Table 4 and Table 5 present the results of the predictive models for the presence of risk of depression, anxiety, and stress. The three predictive models included gender, age, location, physical well-being, psychological well-being, autonomy and parent relationship, social support and peers, school environment, type of displacement, self-concept barriers, motivation and interests, social support barriers, task incompatibility, and PAQ-A scale score. The results show that significant predictors of depression risk (Table 3) are age (OR = 0.82), living in an urban area (OR = 1.70), autonomy and relationship with parents (OR = 0.96), school environment (OR = 0.95), self-concept barrier (OR = 1.49), motivation and interests (OR = 1.75), and total PAQ-A score (OR = 1.55). The significant prediction model for anxiety risk (Table 4) presents the following predictors: living in an urban area (OR = 2.08), physical well-being (OR = 0.96), autonomy and relationship with parents (OR = 0.95), self-concept barrier (OR = 1.51), motivation and interests (OR = 1.82), social support (OR = 1.94), and total PAQ-A score (OR = 1.94). Finally, the predictive model for stress risk (Table 5) was made up of the following: age (OR = 0.80), physical well-being (OR = 0.95), psychological well-being (OR = 1.04), non-active transportation to school (OR = 1.72), self-concept barrier (OR = 1.41), motivation and interests (OR = 1.41), and total PAQ-A score (OR = 2.11). All models showed adequate fit according to the Hosmer–Lemeshow test (*p* > 0.05). In addition, the VIF values of the included variables were less than 2, indicating low collinearity among the predictors.

## 4. Discussion

The results indicate a high prevalence of risk of depression (54.4%), anxiety (63%), and stress (42.2%) among Chilean adolescents, which is consistent with a recent study that examined these post-pandemic variables in the country [4]. This situation can be interpreted using multifactorial models of adolescent mental health, which emphasize the interplay between developmental changes, cognitive-emotional processes, and social–environmental stressors in shaping emotional well-being. In Chile, prolonged school closures during the pandemic, coupled with socioeconomic and educational inequalities, provide a coherent context to explain the elevated prevalence rates observed. This interpretation aligns with recent evidence showing that school closures disrupted peer interaction, learning opportunities, and mental health trajectories, particularly among adolescents from disadvantaged settings, and worsened emotional well-being in contexts marked by structural inequities [31]. These results reveal an alarming situation, given that the prevalence of mental health symptoms in the country is significantly higher than in developed countries, where rates range between 20% and 30% [32]. The social determinants of health framework support this interpretation, as structural inequities and community-level disparities may exacerbate the vulnerability of Chilean adolescents to poor mental health outcomes [4]. The worrying increase in prevalence could be a consequence of the pandemic, during which Chile experienced one of the longest periods of school closures worldwide, altering adolescents’ behaviors by reducing peer interaction and leading to excessive use of technological devices [33,34].

On the other hand, sex-based differences were observed, with women experiencing higher levels of depression, anxiety, and stress. However, only in depression and stress were these differences statistically significant. Some studies have explored these differences in greater depth, showing that being a woman increases the likelihood of mental health symptoms [35], which may be attributed to differences in hormonal regulation during adolescence, influencing mood and emotional regulation [36,37]. Empirical studies have consistently shown that female adolescents often experience higher vulnerability to internalizing symptoms, in part due to differences in access to external psychosocial supports and heightened internal pressures [38].

Several risk factors can affect the mental health of adolescents, notably the family environment, peer relationships, sleep habits, physical inactivity, and unhealthy eating habits [39,40]. Based on this idea, the study results highlight some factors that may influence the high risk levels of participants, such as sports participation (66.1%) and physical activity levels (2.26 ± 0.77). On the one hand, a curious result is the high participation of adolescents in sports activities (66.1%), which is higher than the result established in a study reporting that 43% of adolescents claim to participate in sports activities [41]. With regard to self-reported physical activity, only 17.1% are categorized as having a high level of physical activity, which represents the active category suggested by the World Health Organization physical activity recommendations [42]. This result is lower than that reported in the latest National Survey on Physical Activity and Sport, where 39.5% are active [8]. The lack of consistency between high participation in sports, low levels of physical activity, and high prevalence of mental health risk may be due to a poor understanding of the terminology used to describe participation in sports, where simple physical activity is often confused with practicing a sport [43]. The low percentage of active adolescents is consistent with the high risk of depression, anxiety, and stress, as lower levels of physical activity have been shown to be associated with an increased likelihood of mental health problems [35,39]. From a cognitive-behavioral perspective, discrepancies between perceived and actual activity levels may undermine self-efficacy and intrinsic motivation, further affecting emotional regulation and resilience.

Whit respect to HRQoL, much of the recent evidence in school populations measures the impact of the pandemic on the quality of life of adolescents [44], highlighting the need to further investigate its effects on their physical and mental well-being. In this study, lower quality of life scores were observed across all dimensions, consistent with post-pandemic findings [44,45]. It has been shown that decreased quality of life values in school-age children can directly influence a child’s overall development, affecting their physical, mental, and social development, level of happiness, and academic performance [46].

When analyzing HRQoL by risk of depression anxiety, and stress, the results showed lower scores across all dimensions in adolescents at risk of mental health problems, consistent with evidence showing that depression [47], stress [48] and anxiety [49] negatively impact adolescents’ HRQoL [50]. Specific analysis of the HRQoL dimension revealed those adolescents who were not at risk obtained higher scores in all HRQoL dimensions. A systematic review confirmed these results, finding a significant negative association between HRQoL, depression, and anxiety, while calling for further research into the specific HRQoL dimensions involved [51]. Depression, anxiety, and stress in school-age children have been widely studied [52,53], and are known to be associated with physical health, social relationships, and academic performance [52], all of which are dimensions of HRQoL.

The analysis of the HRQoL dimension shows similar results for depression, anxiety, and stress, with autonomy and relationship with parents, as well as school environment, showing the largest score differences. For depression, adolescents not at risk scored +6.82 points higher in autonomy and relationship with parents and +6.87 in school environment; for anxiety, these differences were +7.07 and +5.02, respectively; and for stress, +6.89 and +5.02. These results may be explained by evidence showing that depression, anxiety, and stress are significantly associated with a hostile, negative, or disconnected parenting style, directly affecting the child–parent relationship [54]. Similarly, the school environment has been shown to influence mental health, with a better socio-educational climate being associated with a lower risk of developing depression and anxiety symptoms [55].

Barriers to physical activity also showed a clear pattern: students at risk of mental health problems scored higher in self-concept, motivation and interests, social support, and task incompatibility barriers, which hinder participation in physical activity [56]. The self-concept barrier is related to body image concerns, which can negatively affect motivation and participation in physical activity [57]. Motivation and interests, social support, and task incompatibility barriers have also been shown to negatively affect adolescents’ mental health, especially among those with low physical activity levels [58,59]. Interestingly, despite statistically significant differences in perceived barriers, PAQ-A scores were similar between adolescents with and without mental health risk, suggesting that self-reported physical activity instruments may not discriminate actual activity levels [60], highlighting the need to use accelerometry in future studies [61].

The regression analysis highlighted several interconnected factors that influence the risk of depression, anxiety, and stress among Chilean adolescents, pointing to the combined influence of sociodemographic characteristics, health-related quality of life dimensions, and perceived barriers to physical activity. Rather than focusing on isolated statistical outputs, these findings suggest that living in urban environments [62], experiencing lower autonomy and weaker relationships with parents [54], reporting poorer school environments, and perceiving greater personal and social barriers to physical activity converge to heightened vulnerability to mental health problems [55,57]. In contrast, higher physical well-being [63], stronger social support [34], and regular physical activity [39] appear to buffer against emotional distress. This holistic interpretation underscores that adolescent mental health is shaped by the interaction between individual behaviors, interpersonal relationships, and contextual conditions, which together determine risk or resilience in this population. These patterns align with international evidence showing that supportive family dynamics [54], inclusive school climates [55], and accessible opportunities for physical activity are protective factors, while environmental stressors and social isolation exacerbate mental health challenges [34].

The study has some limitations. The sampling was non-probabilistic and based on convenience, which limits the generalizability of the findings. Another limitation is the absence of socioeconomic differentiation between schools, which, in the Chilean context, could influence the risk of mental health symptoms [64]. Self-administered questionnaires may also introduce desirability and interpretation biases. Although these instruments have demonstrated validity and reliability in adolescent populations, they remain susceptible to memory biases, social desirability, and subjective interpretation, potentially affecting the accuracy of the data collected. The reported prevalence rates should be interpreted with caution due to the use of screening tools and categorical thresholds, which may overestimate actual prevalence. Additionally, the omission of sports participation as an independent variable limits the ability to assess its specific contribution as a dimension of physical activity. Future studies should incorporate this factor into regression models. Finally, for a better characterization of the effects of lifestyle on psychological well-being, other sedentary behaviors (e.g., sedentary time, screen time) should have been evaluated, so it is recommended that future studies include them.

As strengths, this study addresses an underexplored area, since much of the evidence focuses on adult populations. The findings may inform the development of interventions aimed at promoting physical activity and mental health, taking into account the multiple variables that influence these outcomes. Moreover, this is one of the few national studies to link specific dimensions of HRQoL and perceived barriers to physical activity with adolescent mental health, providing context-specific data that could enhance the effectiveness of future school-based mental health promotion initiatives.

## 5. Conclusions

The present study identified a high prevalence of depressive, anxiety, and stress symptoms among Chilean adolescents, underscoring the need for preventive strategies that address mental health through integrated and context-specific approaches. The findings extend current knowledge by demonstrating that the co-occurrence of low physical activity levels, reduced autonomy and quality of relationships with parents, unfavorable school environments, and perceived barriers—particularly low self-concept and limited motivation—significantly contribute to vulnerability to mental health problems in this population. These results suggest that effective interventions should not be limited to promoting physical activity, per se, but should also target modifiable psychosocial determinants identified in the predictive models, including perceived competence, body image concerns, and the quality of interpersonal relationships within both school and family contexts.

Given that some determinants, such as urban living conditions and socioeconomic disparities, extend beyond the influence of the school system, a comprehensive response will require coordinated action across educational, community, and policy domains. Such multi-level strategies, tailored to the sociocultural realities of Chile, may enhance both the effectiveness and sustainability of adolescent mental health promotion efforts.

## Figures and Tables

**Table 1:** Characteristics of participants.

Variable	All (n = 351)	Male (n = 170)	Female (n = 181)	*p*	d
**Age**	15.75 ± 1.47	15.84 ± 1.44	14.64 ± 1.49	0.21	0.13
**Practices sports (yes)**	232 (66.1%)	140 (82.4%)	92 (50.8%)	00.00	
**Location**					
Urban	170 (48.4%)	99 (58.2%)	107 (59.1%)	0.87	
Rural	181 (51.6%)	71 (41.8%)	74 (40.9%)		
**Physical activity score**	2.26 ± 0.77	2.51 ± 0.81	2.02 ± 0.65	0.00	0.67
**Barriers to physical activity**					
Self-concept	2.44 ± 1.11	2.24 ± 1.03	2.63 ± 1.14	0.00	−0.36
Motivation and interests	2.30 ± 0.97	2.11 ± 0.911	2.48 ± 0.99	0.00	−0.39
Social support	2.33 ± 1.01	2.24 ± 1.05	2.41 ± 0.98	1.2	−0.17
Incompatibility of tasks	2.66 ± 1.00	2.58 ± 1.05	2.74 ± 0.95	1.4	0.16
**Health-related quality of life**					
Physical well-being	40.17 ± 8.67	43.96 ± 8.68	36.70 ± 7.11	0.00	0.90
Psychological well-being	37.03 ± 6.25	37.86 ± 7.03	36.24 ± 5.32	0.02	0.26
Autonomy and parent relation	47.35 ± 12.42	48.91 ± 13.11	45.88 ± 11.54	0.02	0.25
Social support and peers	53.08 ± 12.20	53.12 ± 12.29	53.04 ± 12.14	0.95	0.00
School environment	47.50 ± 10.77	48.87 ± 10.53	46.22 ± 10.87	0.02	0.25
**Mental health**					
Risk of depression	191 (54.4%)	83 (48.8%)	108 (59.7%)	0.04	
Risk of anxiety	221 (63%)	100 (58.8%)	121 (66.9%)	0.12	
Risk of stress	148 (42.2%)	62 (36.5%)	86 (47.5%)	0.04	

Variable names in bold. Values of <0.1, 0.1–0.29, 0.3–0.49 and ≥0.5 were considered trivial, small, moderate, or large, respectively.

**Table 2:** Dimensions of health-related quality of life, barriers to physical activity, and physical activity score based on risk of depression, anxiety, and stress.

Variable	Depression				Anxiety				Stress			
	At Risk	Not at Risk	*p*	d	At Risk	Not at Risk	*p*	d	At Risk	Not at Risk	*p*	d
**Physical activity score**	2.26 ± 0.815	2.26 ± 0.718	0.98	0.00	2.28 ± 0.789	2.22 ± 0.742	0.438	0.00	2.34 ± 0.827	2.20 ± 0.725	0.10	−0.18
**Barriers to physical activity**												
Self-concept	2.81 ± 1.11	2.01 ± 0.936	0.00	−0.78	2.71 ± 1.13	1.98 ± 0.898	0.000	−0.78	2.85 ± 1.16	2.14 ± 0.964	0.00	−0.67
Motivation and interests	2.60 ± 0.947	1.95 ± 0.87	0.00	−0.72	2.49 ± 0.938	1.99 ± 0.948	0.000	−0.72	2.61 ± 1.00	2.09 ± 0.887	0.00	−0.55
Social support	2.57 ± 1.02	2.04 ± 0.95	0.00	−0.54	2.45 ± 1.01	2.11 ± 1.01	0.003	−0.54	2.58 ± 1.07	2.15 ± 0.941	0.00	−0.43
Incompatibility of tasks	2.73 ± 0.994	2.58 ± 1.02	0.18	−0.14	2.75 ± 1.00	2.51 ± 0.999	0.031	−0.14	2.85 ± 0.996	2.52 ± 0.997	0.00	−0.33
**Health-related quality of life**												
Physical well-being	38.18 ± 8.30	42.54 ± 8.53	0.00	0.52	38.69 ± 8.44	42.67 ± 8.51	0.000	0.52	37.86 ± 8.06	41.84 ± 8.73	0.00	0.47
Psychological well-being	36.27 ± 6.20	37.93 ± 6.22	0.01	0.27	36.65 ± 6.25	37.67 ± 6.23	0.140	0.27	36.77 ± 6.54	37.77 ± 6.05	0.51	0.07
Autonomy and parent relation	44.24 ± 12.42	51.06 ± 11.40	0.00	0.57	44.73 ± 12.20	51.80 ± 12.10	0.000	0.57	43.37 ± 12.23	50.26 ± 11.77	0.00	0.58
Social support and peers	52.16 ± 12.73	54.18 ± 12.58	0.12	0.17	52.25 ± 12.20	54.49 ± 12.10	0.097	0.17	51.57 ± 12.94	54.18 ± 11.53	0.05	0.22
School environment	44.37 ± 10.45	51.24 ± 9.96	0.00	0.68	45.64 ± 10.65	50.66 ± 10.27	0.000	0.67	44.60 ± 10.61	49.62 ± 10.42	0.00	0.48

**Table 3:** Binary logistic regression model predicting the risk of depression.

	R2 Cox y Snell						95% C.I. for EXP (B)	
		β	DE.	Wald	*p*	OR	Lower	Higher
**Age**	0.25	−0.18	0.09	4.09	0.04	0.82	0.69	0.99
**Location (Urban)**		0.53	0.26	4.04	0.04	1.70	1.01	2.86
**Independence and relationship with parents**		−0.03	0.01	9.39	0.00	0.96	0.94	0.98
**School environment**		−0.04	0.01	8.97	0.00	0.95	0.93	0.98
**Self-concept**		0.37	0.15	5.95	0.01	1.44	1.07	1.95
**Motivation and interests**		0.56	0.18	9.72	0.00	1.75	1.23	2.50
**Physical activity score**		0.43	0.17	6.09	0.01	1.55	1.09	2.19
**Constant**		3.43	1.89	3.29	0.06	31.10		

**Table 4:** Binary logistic regression model predicting anxiety risk.

	R2 Cox y Snell						95% C.I. for EXP (B)	
		β	DE.	Wald	*p*	OR	Lower	Higher
**Location (urban)**	0.19	0.73	0.25	8.00	0.00	2.08	1.25	3.45
**Physical well-being**		-0.03	0.01	3.61	0.05	0.96	0.93	1.00
**Autonomy and parent relation**		-0.04	0.01	17.4	0.00	0.95	0.93	0.97
**Self-concept**		0.41	0.16	6.05	0.01	1.51	1.08	2.10
**Motivation and interests**		0.60	0.21	8.09	0.00	1.82	1.20	2.76
**Social support**		-0.33	0.17	3.42	0.06	0.71	0.50	1.02
**Physical activity score**		0.66	0.19	11.08	0.00	1.94	1.31	2.86
**Constant**		0.72	0.99	0.52	0.46	2.05		

**Table 5:** Binary logistic regression model predicting stress risk.

	R2 Cox y Snell						95% C.I. for EXP (B)	
		β	DE.	Wald	*p*	OR	Lower	Higher
**Age**	0.21	−0.22	0.08	6.29	0.01	0.80	0.67	0.95
**Physical well-being**		−0.05	0.02	5.97	0.01	0.95	0.91	0.99
**Psychological well-being**		0.04	0.02	4.53	0.03	1.04	1.00	1.09
**Autonomy and parent relation**		−0.05	0.01	19.50	0.00	0.94	0.92	0.97
**Passive transport home**		0.54	0.31	2.92	0.08	1.72	0.92	3.20
**Self-concept**		0.30	0.15	4.08	0.04	1.35	1.00	1.81
**Motivation and interests**		0.35	0.17	4.11	0.04	1.41	1.01	1.99
**Physical activity score**		0.75	0.19	14.35	0.00	2.11	1.43	3.12
**Constant**		2.16	1.74	1.53	0.21	8.72		

## Data Availability

Data are contained within the article.
